# Spatial distribution of stunting and wasting in 6–59 months children in Nepal: analysis using a Bayesian distributional bivariate probit model

**DOI:** 10.1017/jns.2023.9

**Published:** 2023-02-23

**Authors:** Richa Vatsa, Umesh Ghimire, Suman Sapkota, Raj Kumar Subedi

**Affiliations:** 1 Central University of South Bihar, SH-7, Gaya Panchanpur Road, Karhara, Post. Fatehpur, Gaya, Bihar 824236, India; 2 School of Public Health, University of Minnesota, 420 Delaware St SE, Minneapolis, MN 55455, USA; 3 Department of Child and Adolescent Health and Maternal Care, School of Public Health, Capital Medical University, Beijing 100069, China; 4 Bhaskar Tejshree Memorial Foundation, Kathmandu 44600, Nepal

**Keywords:** Bivariate probit model, Malnutrition, Nepal, Stunting, Wasting, BMI, body mass index, CrI, credible interval, DHS, Demographic and Health Survey, HDI, Human Development Index, IYCF, Infant and Young Child Feeding, LMICs, low- and middle-income countries, NDHS, Nepal Demographic and Health Survey, PSUs, primary sampling units, WHO, World Health Organization

## Abstract

The combined burden of stunting and wasting in children under five years is a serious public health concern. The present study aimed to estimate the joint burden of stunting and wasting among children aged 6–59 months and explore its spatial variation across Nepal. The 2016 Nepal Demographic and Health Survey data was used to study acute and chronic childhood malnutrition. A Bayesian distributional bivariate probit geoadditive model was designed to study the linear association and geographical variation of stunting and wasting among 6–59 months, children. Child-related factors such as low birth weight, fever in the last 2 weeks preceding the survey and fourth or greater birth order were associated with a higher likelihood of stunting. The likelihood of a child being stunted was significantly less in the wealthiest households, having improved toilet facilities, and if mothers were overweight. Children from severely food insecure households were significantly more likely, and children from poorer households were significantly less likely to suffer both acute and chronic malnutrition simultaneously. Results from spatial effect showed that children from Lumbini and Karnali had a higher burden of stunting, and the likelihood that achild would have been wasted was significantly higher in Madhesh and Province 1. Immediate nutritional efforts are vital in low-income and severely food insecure households to lessen the risk of stunting and wasting in under children. Disproportionate geographic variations in stunting and wasting warrant sub-regional-specific nutrition intervention to achieve nutrition targets and reduce the burden of childhood malnutrition across the country.

## Background

Adequate nutrition is crucial for the physical and cognitive development of children^(1,2)^. Apart from optimal diet, a child's overall development is based on immunisation, growth monitoring, care and support. Childhood nutrition is an essential indicator of a country's social and economic development^(3,4)^. Nevertheless, child malnutrition continues to be a major health issue, accounting for nearly half of all under-five deaths globally^(4,5)^. High illiteracy, gender-based violence, low decision-making among women, fading agricultural practices and a lack of job opportunities are enabling factors of childhood undernutrition in Nepal^(6,7)^.

According to the Nepal National Micronutrient Status Survey 2016, 35 % of Nepalese children aged 6–59 months were stunted (short height-for-age), a condition caused by chronic malnutrition^(8)^. Similarly, 11⋅3 % of children aged 6–59 months experienced wasting (low weight-for-height), an indicator of acute malnutrition caused by a lack of nutritional demands owing to sickness or infection^(9,10)^. South Asian countries have the world's highest childhood wasting rates^(10)^. Children suffering from malnutrition are at a much-increased risk of infection-related death. Undernutrition increases the frequency and severity and delays the recovery of several infections^(11)^. Nepal has achieved a notable decline in childhood undernutrition and other Millennium Development Goals (MDGs) indicators during the last several decades, as well as a significant reduction in under-five death rates^(12)^. The stunting rates have declined from 57 % in 2001 to 36 % in 2016, and several studies have highlighted the success stories of stunting reduction in Nepal^(13)^; however, wasting remained stagnant over the same period^(14)^. Moreover, disparities are widespread in reducing undernutrition across different geographic regions, socioeconomic groups and ethnicity. The Sustainable Development Goals (SDGs) aim to reduce wasting below 5 % by 2025, which requires regional-level policies and programs intervention^(15)^. The target of the Government of Nepal Multi-sector Nutrition Plan II was to level off stunting at 28 % by 2022 and reduce wasting from 9⋅7 % in 2016 to 7 % in 2022^(12)^.

Taming malnutrition and understanding its determinants requires critical scrutiny at different geographic levels. Regression modelling is a standard method that most studies use to explore childhood malnutrition, considering the indicators as univariate outcomes. The assumptions of regression analyses are based on homoscedasticity and consider no relationship between the nutritional outcomes. On the other hand, outcomes are associated with the conditional mean of the distribution considered for the model. Different nutrition-related indicators are measured from the same children at the same time point in the survey; hence, it is liable that an association exists between the other indicators.

The present study applies a distributional bivariate probit model to explore the association between acute and chronic malnutrition and other factors^(16)^. The inclusion of structured additive predictor into the model permits linking spatial and other covariates to the means and correlations between the undernutrition indicators^(17,18)^. The categorical predictors under the study were assumed to have a linear effect; the effects of metrical covariates were considered non-linear and modelled non-parametrically. The district of a child's residency was added as a geographical covariate in the model to investigate spatial variation in the association between stunting and wasting among children in Nepal. A district-level assessment could provide estimates for the regional heterogeneity of malnutrition, which could aid in reducing its prevalence at the national level.

## Methods

### Data source

Data from the 2016 Nepal Demographic and Health Survey (NDHS) was extracted for this study. The 2016 NDHS was executed under the flagship of the United States Agency for International Development (USAID). The DHS program, which is usually undertaken every 5 years, aids in gathering key demographic and health-related information in many developing nations. Interviews of Demographic and Health Surveys (DHS) occur in households with women of reproductive age (WRA) (15–49 years) and their children born in the 5 years before the survey. In the 2016 NDHS, a two-stage sample strategy was used in rural regions, and a three-stage sampling approach was used in urban areas. At first, stratified random sampling was employed to select 383 clusters across the country based on the population proportional to size method. Secondly, thirty households were randomly selected from each cluster using equal probability systematic sampling. Further information about the survey can be obtained from the respective reports^(19)^.

The information of 2061 live children aged 6–59 months whose mothers were not pregnant and had their body mass index (BMI) measured at the time of the interview was extracted for this study. The data consisted of information regarding children's household characteristics, maternal characteristics, nutritional status, illness status and birth characteristics. After removing missing data (*n=* 66) on children's stunting and wasting status, 1995 samples were considered for the final analysis.

### Study outcomes

Two binary variables indicating children's nutritional status, stunting and wasting were used as the primary outcomes in this study. These variables were obtained from height and weight measurements of children aged 6–59 months. Stunting relates to low height-for-age measuring linear growth retardation and cumulative growth deficits. It was calculated with binary coding: 1 if the *z*-score of height-for-age below −2 standard deviations from the World Health Organization Child Growth Standards median, and 0 otherwise. On the other hand, wasting refers to low weight-for-height, indicating failure to receive adequate nutrition. Wasting was coded as 1 for the *z*-score of height-for-age below −2 standard deviations from the median of the World Health Organization Child Growth Standards and 0 otherwise. Malnourished, stunted or wasted children were coded 1 and 0 otherwise^(20)^.

### Explanatory variables

Explanatory variables were grouped under household characteristics, maternal characteristics, child characteristics related to infection and illnesses, and their feeding practices. The variables were further categorised as categorical, continuous or spatial. Continuous variables included the mother's age in completed years and the child's age in completed months. Mother's place of residence was categorised as urban and rural. Households having either flush or pour-flush toilets to a piped water system, septic tank or pit latrine, ventilated improved pit latrine, pit latrine with a slab or composting toilet, and do not share this facility with other households were categorised into improved toilet facilities. The wealth of households was divided into five ordinal groups ranging from poorest to richest. The wealth score was calculated using principal component analysis and the types of consumer items owned by the households, along with housing characteristics^(21)^. The number of household members was divided into two categories: those with five or fewer family members and those with five or more. Household food insecurity was assessed using the nine questions from the Household Food Insecurity Access Scale. Based on the responses, the food insecurity status of households was classified into four groups: secure, mildly insecure, moderately insecure and severely insecure^(22)^. Mother's education status includes no education, primary, secondary and higher education. The mother's working status was classified as yes or no, depending on her working status in the 12 months before the survey. The current breastfeeding status was used as a dichotomous variable (0/1). Mother's BMI was calculated using two anthropometric indices, height and weight, measured by standardised scales. Four categories of BMI were included: underweight (<18⋅5 kg/m^2^), normal (18⋅5–23⋅0 kg/m^2^), overweight (23⋅0–27⋅5 kg/m^2^) and obesity (>27⋅5 kg/m^2^)^(23)^. Mothers were asked whether children experienced fever and/or cough in the past 2 weeks preceding the survey. The responses were recorded as yes or no. Three groups of child's size at birth (large, average and small) were extracted from a written child health record or reported by the mother. Similarly, the birth orders of a child were categorised as the first, second or third, and fourth or higher. The district of mother's residence was used to explore the spatial variation of child malnutrition^(19)^. The rest of the study variables were considered categorical and analysed together with the continuous and spatial variables to assess possible effects on the malnutrition status of children. All the categorical explanatory variables are listed in Table 1 with their frequency distributions.

**Table 1.** Distribution of nutritional status of children under different study variables and their association through *P*-value

Variable	Total	Stunting %	*P*-value	Wasting %	*P*-value
	*N* (%)				
Household characteristics					
Place of residence			<0⋅001		0⋅5
Urban	1120 (56⋅1)	34⋅6		10⋅1	
Rural	875 (43⋅9)	43⋅8		9⋅1	
Household wealth			<0⋅001		0⋅2
Poorest	493 (24⋅7)	53⋅6		8⋅9	
Poorer	436 (21⋅9)	42⋅0		9⋅4	
Middle	428 (21⋅4)	35⋅1		10⋅5	
Richer	403 (20⋅2)	32⋅5		11⋅9	
Richest	235 (11⋅8)	18⋅3		6⋅4	
Size of household			0⋅05		0⋅1
≤5	998 (50⋅0)	36⋅5		8⋅6	
>5	997 (50⋅0)	40⋅8		10⋅7	
*Food insecurity*			<0⋅001		0⋅6
Food secure	797 (39⋅9)	30⋅2		8⋅9	
Mildly food insecure	453 (22⋅7)	38⋅4		10⋅8	
Moderately food insecure	544 (27⋅3)	46⋅7		9⋅2	
Severely food insecure	201 (10⋅1)	50⋅8		11⋅4	
Toilet facility			<0⋅001		0⋅002
Improved	1527 (76⋅5)	35⋅8		8⋅5	
Unimproved	468 (23⋅5)	48⋅1		13⋅5	
Maternal characteristics					
Working status of mother			<0⋅001		0⋅003
Working	1123 (56⋅3)	41⋅8		7⋅9	
Not working	872 (43⋅7)	34⋅6		11⋅9	
Educational status of mother			<0⋅001		0⋅01
No education	677 (33⋅9)	48⋅2		12⋅6	
Primary	368 (18⋅5)	39⋅7		7⋅9	
Secondary	655 (32⋅8)	34⋅1		9⋅0	
Higher	295 (14⋅8)	25⋅8		6⋅8	
Mother's BMI			<0⋅001		0⋅002
Underweight	375 (18⋅8)	45⋅6		13⋅6	
Normal	1070 (53⋅6)	40⋅8		10⋅1	
Overweight	422 (21⋅2)	30⋅3		6⋅4	
Obese	128 (6⋅4)	27⋅3		5⋅5	
Child characteristics					
Sex of child			1		0⋅6
Female	934 (46⋅8)	38⋅8		10⋅1	
Male	1061 (53⋅2)	38⋅6		9⋅2	
Size at birth			<0⋅001		0⋅03
Large	312 (15⋅6)	34⋅3		5⋅8	
Average	1332 (66⋅8)	36⋅7		10⋅1	
Small	351 (17⋅6)	49⋅9		11⋅4	
Birth order			<0⋅001		0⋅2
First	759 (38⋅0)	32⋅4		9⋅5	
Second or third	898 (45⋅0)	39⋅4		8⋅8	
Fourth or greater	338 (17⋅0)	50⋅6		12⋅4	
Infection and illness					
Fever in 2 weeks preceding the survey			0⋅9		0⋅009
No	1558 (78⋅1)	38⋅5		8⋅7	
Yes	437 (21⋅9)	39⋅1		13⋅0	
Cough in 2 weeks preceding the survey			0⋅2		0⋅03
No	1572 (78⋅8)	39⋅4		8⋅9	
Yes	423 (21⋅2)	35⋅9		12⋅5	
Diarrhoea in 2 weeks preceding the survey			0⋅7		0⋅3
No	1853 (92⋅9)	38⋅8		12⋅7	
Yes	142 (7⋅1)	36⋅6		9⋅4	
Feeding practices					
Currently breastfeeding			0⋅1		<0⋅001
Yes	1188 (59⋅5)	37⋅3		12⋅2	
No	807 (40⋅5)	40⋅6		6⋅0	

### Statistical analysis

We employed a bivariate probit distribution regression model^(17,24)^ to jointly fit the binary variables – stunting and wasting, accounting for their correlation and estimating their association with the considered covariates. Let, for an *i*th child, *Y*_*i*1_ and *Y*_*i*2_ denote stunting and wasting as binary response variables, and 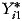 and 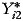 refer to corresponding latent variables, defined such that, for *j*  = 1,2
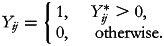


In other words, a child is considered stunted or wasted if the values of the corresponding latent variables are positive. Furthermore, let (*Y*_*i*1_, *Y*_*i*2_)^′^ denote a vector of the two correlated binary response variables. The bivariate probit distribution of (*Y*_*i*1_, *Y*_*i*2_)^′^ conditional on the considered covariates can be perceived from the joint distribution of the latent variables 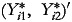.

For the ease of refering the terms ahead, subscript i has been deleted from the notations. Let *X* denote the vector of categorical covariates, *u*, continuous covariates, *w*, and spatial covariates, *s*, under study such that *X* = (*u*, *w*, *s*)^′^. The latent variables 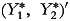, conditional on *X*, are assumed to jointly follow a bivariate normal distribution, i.e., 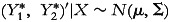. The term *μ* = (*μ*_1_, *μ*_2_)^′^ is a vector of means of latent variables 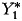 and 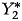 for stunting and wasting, respectively; and 
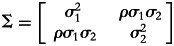
 is the covariance matrix. The term *ρ* is the correlation coefficient between the latent variables; whereas the variances 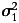 and 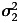
of 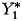 and 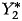, respectively, are assumed as 1 in diagonal places of Σ to enable identifiability.

We consider the distributional probit model of Klein *et al.*^(25)^, which allows capturing the effects of covariates on all the model parameters. Let a vector of the parameters *μ*_1_, *μ*_2_ and *ρ* be denoted by *θ* = (*θ*_1_ = *μ*_1_, *θ*_2_ = *μ*_2_, *θ*_3_ = *ρ*). Following the framework of structured additive distribution regression of Klein *et al.*^(25)^, the parameter *θ*_*k*_ can be associated with the covariates through geoadditive predictors 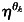 through appropriate link functions ensuring the restrictions on the parameter space such that 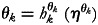
^(25)^.

The link function 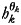 in the case of mean parameters *μ*_1_ and *μ*_2_ are assumed as identity, whereas to the model correlation coefficient, *ρ*, Fisher *z*-transformation is considered. The predictor,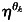, linked to the *k*th parameter can be written in a matrix form as, 
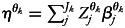
, where 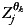 stands for design matrices found by basis function expansion. The terms 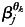 are the vectors of regression coefficients that are assigned an improper Gaussian prior, as
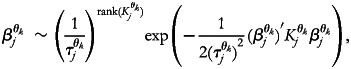
where the term 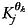 refers to a prior precision matrix, and 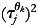 are variance parameters to control the smoothness of the prior and are assigned with inverse gamma hyperprior.

The predictor 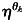 may be defined with the general form, 
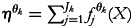
, where the functions 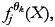 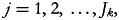 refer to different effects of different covariates. For example, 
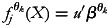
 relate to linear effects of vector of categorical covariates *u* with linear effect parameters 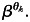 In this linear case, the design matrix 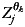 simply be the vector *u*. Similarly, smooth functions 
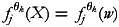
 stand for non-linear effects of continuous covariate vectors *w*. Furthermore, the function 
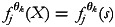
 accounts for spatial effects for discrete spatial units, *s*.

The linear effect parameters were assumed with a flat non-informative prior, i.e., 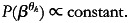 For the non-linear smooth functions 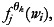 Bayesian P-spline priors^(26)^ were considered with the design matrix 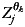 comprising of D B-spline basis functions found from piecewise polynomials defined on equidistant grid of knots. According to the literature, we employed cubic B-splines based on twenty equidistant knots, which provide sufficient flexibility to capture complex non-linearity. We considered a second-order random walk prior over the basis coefficients and an inverse gamma hyperprior over the variance parameters of the prior^(27)^. Furthermore, a Gaussian Markov random field (GMRF) prior^(28)^ was utilised to model spatial effects, 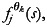 where *s* are discrete spatial locations on a geographical map. In case of spatial effects, the design matrix 
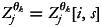
 are indicator functions with value 1 if the *i*th observation relates to the location *s*, or with values 0 otherwise. Thus, the spatial effects 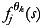 are considered as equal to the spatial coefficients 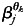. GMRF considers adjacent spatial locations as neighbours if they share a common boundary and thus are correlated. Assuming *N*(*s*) as the vector of neighbours of location, *s*, the conditional distribution of 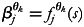 given the spatial effects at locations other than *s* is Gaussian with mean parameter as the average of spatial effects of all neighbours of *s*, and variance proportional to number of neighbours of *s*, |*N*(*s*)|. That is, a GMRF prior over 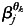 is defined as
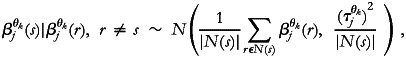
where the smoothness parameter 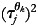 was assumed to follow inverse gamma hyperprior. More details of the Bayesian structured additive models can be found in the literature^(29)^.

The Bayesian estimation of the parameters of the above-mentioned bivariate probit distributional model was achieved via the Metropolis-Hastings algorithm of Markov Chain Monte Carlo (MCMC) techniques, based on iteratively weighted least square (IWLS) as proposed by Klein *et al.*^(25)^. A multicollinearity check was performed between the linear covariates through the variance inflation factor (VIF), however, no multicollinearity issue was found among the covariates as the VIF values obtained were all less than 2⋅5. The analysis was executed using openly available software for inference in structured additive models, BayesX^(29)^ using R-package BayesXSrc^(30)^. For all associations, *P*-values less than 0⋅05 were considered statistically significant.

## Results

Table 1 presents the distribution of children's nutritional status-stunting and wasting under different headings of household characteristics, maternal characteristics, child characteristics, child's infection and illness status, and their nutritional practices. Of the 1995 children, 40⋅7 % were stunted and 9⋅7 % were wasted. Most stunted children belonged to rural (43⋅8 %), poorest (53⋅6 %) and poorer (41⋅97 %) households, whereas the percentage of wasted children was higher among urban (10⋅09 %), middle (10⋅51 %) and richer (11⋅91 %) households. Approximately 51 % of severely food insecure households and 47 % of moderately food insecure households had stunted children. On the other hand, nearly 12 % of severely food insecure households and 11 % of moderately food insecure households had wasted children. Households with unimproved toilet facilities and more than five members had more stunted (48⋅08 % and 40⋅82 %, respectively) and wasted (13⋅46 % and 10⋅73 %, respectively) children. Furthermore, almost 42 % of working mothers, 48 % of those with no education, and 46 % of underweight mothers had stunted children. While nearly 12 % of non-working mothers, 13 % of those with no education and 14 % of underweight mothers had wasted children. Around 50 % of children with small size at birth and 51 % of those in fourth or higher in birth order were found to be stunted. Nevertheless, around 11 % of children with small size at birth and 12 % of those in fourth or higher birth order were found wasted. Nearly 13 % of children who had fever or cough in the last 2 weeks preceding the survey were also wasted. Around 41 % of children who were not currently breastfed were stunted. However, around 12 % of currently breastfed children were wasted.

Table 1 also presents the association of the nutritional status of children with the above-mentioned study variables through the *P*-value obtained from the *χ*^2^ test for independence of attributes. At a 5 % level of significance, stunting status of children was significantly associated with place of residence, toilet facility, household wealth, size of household, food insecurity status of the household, working status of the mother, educational status of mother, mother's BMI, child's size at birth, child's birth order and child's current breastfeeding status. On the other hand, at a 5 % level of significance, the wasting status of children was significantly associated with household toilet facility, mother's working status, educational status and BMI, child's size at birth, child's illness status-fever and cough in 2 weeks preceding the survey, and child's current breastfeeding status. As per the results presented in Table 1, the categorical study variables significantly associated with either stunting or wasting were considered into the model for further analysis.

Table 2 presents the posterior mean and 95 % credible intervals of linear effects of covariates on the mean levels of stunting, wasting and the latent correlation between them. The estimates suggest that likelihood of a child being stunted was significantly less in the richest households and those with improved toilet facilities. On the other hand, children with small birth size, or fourth or higher in birth order, or who had fever in 2 weeks preceding the survey, were significantly more likely to be stunted. Furthermore, children born to underweight mothers were significantly more likely to be stunted or wasted. Children with overweight mothers, on the other hand, had a significantly lower chance of being stunted. Moreover, currently breastfed children were significantly more likely to be wasted. The negative estimates for the latent association between two nutritional status indicators indicate that children cannot be stunted and wasted at the same time, whereas positive estimates imply the opposite. Latent correlation results suggest that children from lower-income families, those born to mothers with primary education and those born with small birth weights were less likely to suffer from both stunting and wasting. On the other hand, children from the severe food insecure households were significantly more likely to suffer from both stunting and wasting.

**Table 2.** Posterior estimates of stunting, wasting and their correlation with 95 % credible intervals

	*μ* (Stunting)		*μ* (Wasting)		*ρ* (correlation)	
	Posterior estimates		Posterior estimates		Posterior estimates	
	Mean	95 % CrI	Mean	95 % CrI	Mean	95 % CrI
Place of residence						
Urban (ref.)	0⋅000		0⋅000		0⋅000	
Rural	0⋅019	(−0⋅047, 0⋅083)	−0⋅057	(−0⋅145, 0⋅028)	−0⋅1	(−0⋅305, 0⋅069)
Household wealth						
Poorest (ref.)	0⋅000		0⋅000		0⋅000	
Poorer	0⋅079	(−0⋅041, 0⋅2)	−0⋅057	(−0⋅23, 0⋅107)	−0⋅473*	(−0⋅911, −0⋅138)
Middle	−0⋅047	(−0⋅166, 0⋅081)	−0⋅043	(−0⋅212, 0⋅112)	−0⋅112	(−0⋅462, 0⋅218)
Richer	−0⋅023	(−0⋅153, 0⋅101)	0⋅131	(−0⋅033, 0⋅299)	0⋅262	(−0⋅084, 0⋅701)
Richest	−0⋅3*	(−0⋅485, −0⋅115)	−0⋅072	(−0⋅325, 0⋅172)	0⋅539	(−0⋅038, 1⋅148)
Size of household members						
>5 (ref.)	0⋅000		0⋅000		0⋅000	
≤5	−0⋅02	(−0⋅081, 0⋅042)	−0⋅046	(−0⋅132, 0⋅037)	0⋅152	(−0⋅033, 0⋅342)
Household food insecurity						
Food secure (ref.)	0⋅000		0⋅000		0⋅000	
Mildly food insecure	−0⋅06	(−0⋅177, 0⋅064)	0⋅01	(−0⋅139, 0⋅159)	−0⋅181	(−0⋅535, 0⋅12)
Moderately food insecure	0⋅07	(−0⋅036, 0⋅172)	−0⋅079	(−0⋅216, 0⋅071)	−0⋅076	(−0⋅373, 0⋅236)
Severely food insecure	0⋅061	(−0⋅088, 0⋅21)	0⋅099	(−0⋅106, 0⋅278)	0⋅869*	(0⋅42, 1⋅463)
Toilet facility						
Unimproved (ref.)	0⋅000		0⋅000		0⋅000	
Improved	−0⋅137*	(−0⋅216, −0⋅061)	−0⋅069	(−0⋅17, 0⋅037)	0⋅016	(−0⋅186,0⋅232)
Educational status of mother						
No education (ref.)	0⋅000		0⋅000		0⋅000	
Primary	−0⋅039	(−0⋅164, 0⋅08)	−0⋅047	(−0⋅213, 0⋅116)	−0⋅384*	(−0⋅767, −0⋅036)
Secondary	0⋅038	(−0⋅067, 0⋅14)	0⋅017	(−0⋅13, 0⋅162)	0⋅182	(−0⋅137, 0⋅501)
Higher	−0⋅083	(−0⋅236, 0⋅063)	−0⋅147	(−0⋅357, 0⋅063)	0⋅17	(−0⋅258, 0⋅635)
Mother's BMI						
Normal (ref.)	0⋅000		0⋅000		0⋅000	
Underweight	0⋅15*	(0⋅021, 0⋅282)	0⋅191*	(0⋅025, 0⋅366)	0⋅02	(−0⋅377, 0⋅442)
Overweight	−0⋅124*	(−0⋅247, −0⋅004)	−0⋅072	(−0⋅249, 0⋅101)	0⋅051	(−0⋅379, 0⋅506)
Obese	−0⋅096	(−0⋅285, 0⋅089)	−0⋅194	(−0⋅522, 0⋅086)	−0⋅158	(−0⋅968, 0⋅649)
Size at birth						
Large (ref.)	0⋅000		0⋅000		0⋅000	
Average	−0⋅063	(−0⋅145, 0⋅022)	0⋅025	(−0⋅094, 0⋅148)	−0⋅095	(−0⋅337, 0⋅13)
Small	0⋅259*	(0⋅149, 0⋅366)	0⋅123	(−0⋅032, 0⋅274)	−0⋅317*	(−0⋅606, −0⋅045)
Birth order						
First (ref.)	0⋅000		0⋅000		0⋅000	
Second or third	0⋅012	(−0⋅071, 0⋅098)	−0⋅065	(−0⋅187, 0⋅049)	0⋅11	(−0⋅117, 0⋅369)
Greater than third	0⋅168*	(0⋅026, 0⋅306)	0⋅011	(−0⋅181, 0⋅197)	0⋅294	(−0⋅057, 0⋅686)
Fever in 2 weeks preceding the survey						
No (ref.)	0⋅000		0⋅000		0⋅000	
Yes	0⋅19*	(0⋅012, 0⋅365)	0⋅082	(−0⋅159, 0⋅303)	−0⋅029	(−0⋅485, 0⋅392)
Cough in 2 weeks preceding the survey						
No (ref.)	0⋅000		0⋅000		0⋅000	
Yes	−0⋅124	(−0⋅298, 0⋅048)	0⋅106	(−0⋅111, 0⋅333)	−0⋅037	(−0⋅489, 0⋅421)
Currently breastfeeding						
No (ref.)	0⋅000		0⋅000		0⋅000	
Yes	0⋅021	(−0⋅065, 0⋅107)	0⋅191*	(0⋅072, 0⋅313)	0⋅148	(−0⋅093, 0⋅41)
*Constant*	−0⋅284*	(−0⋅438, −0⋅115)	−1⋅576*	(−1⋅817, −1⋅37)	0⋅414	(−0⋅063, 0⋅884)

Crl, credible interval, ref., reference.

*Significant at less than 0.05 level of significance.

**Fig. 1.**
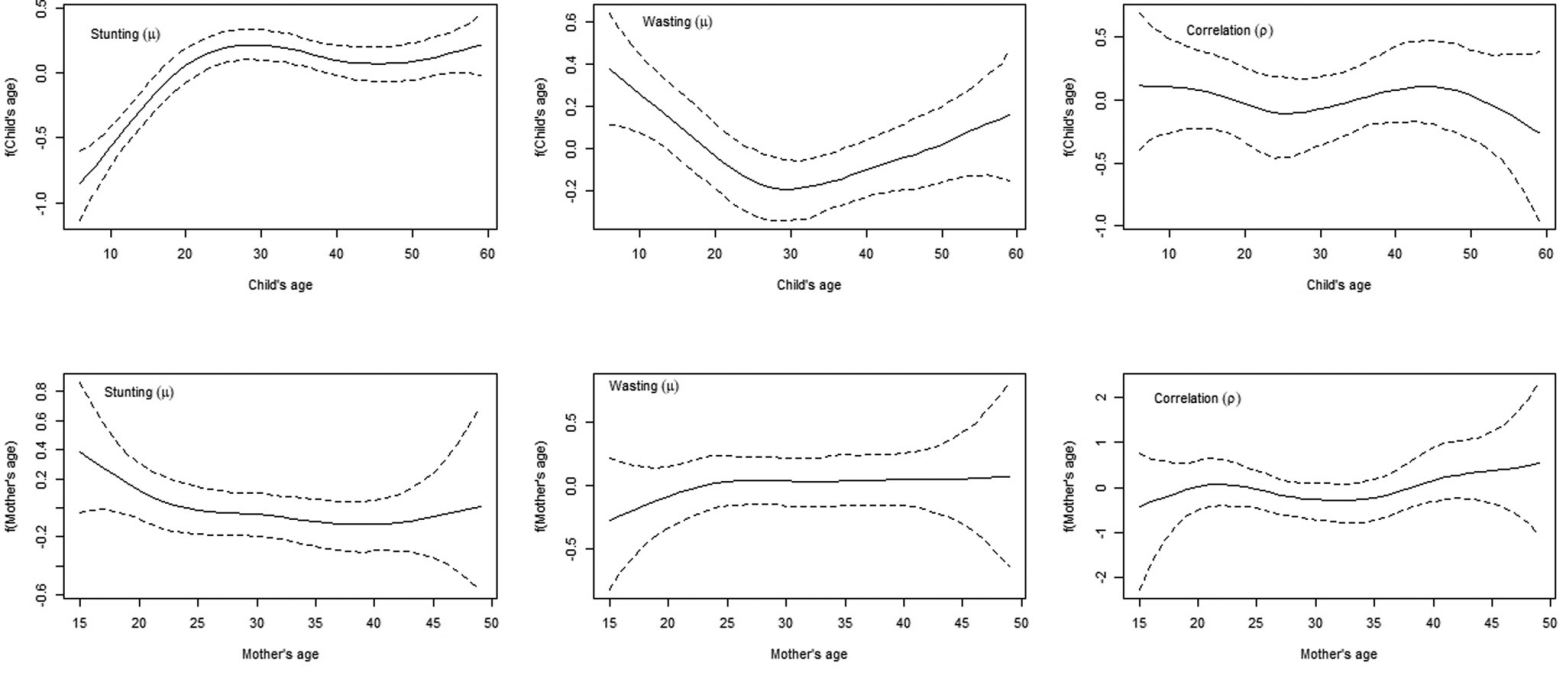
Non-linear effects of child's age and mother's age on stunting (*μ*), wasting (*μ*) and correlation (*ρ*) with posterior mean (solid line) and 95 % credible intervals (dashed lines).

The posterior estimates (mean and 95 % credible intervals) of non-linear effects of covariates – mother's age and child's age are shown in Fig. 1. The results for the mean level of stunting indicate that the likelihood of stunting in children persisted until the age of 29 months; then declined until a child reached the age of 40 months. However, risk of stunting in children dropped dramatically between the ages of 25 and 40 months. The likelihood of stunting in children increased sharply after the mother's age reached 40 years. On the other hand, chances of a child being wasted reduced drastically around the age of 30 months and rapidly increased thereafter. However, it persisted until mothers were 25 years old after which the risks of wasting remained more or less constant. The latent correlation between stunting and wasting declined with child age until 25 months thereafter, it peaked around 45 months and plummeted. Correlation, on the other hand, increased as the mother's age climbed until the age of 22, then declined gradually until the mother was 32 years, then to spiked rapidly again.

**Fig. 2.**
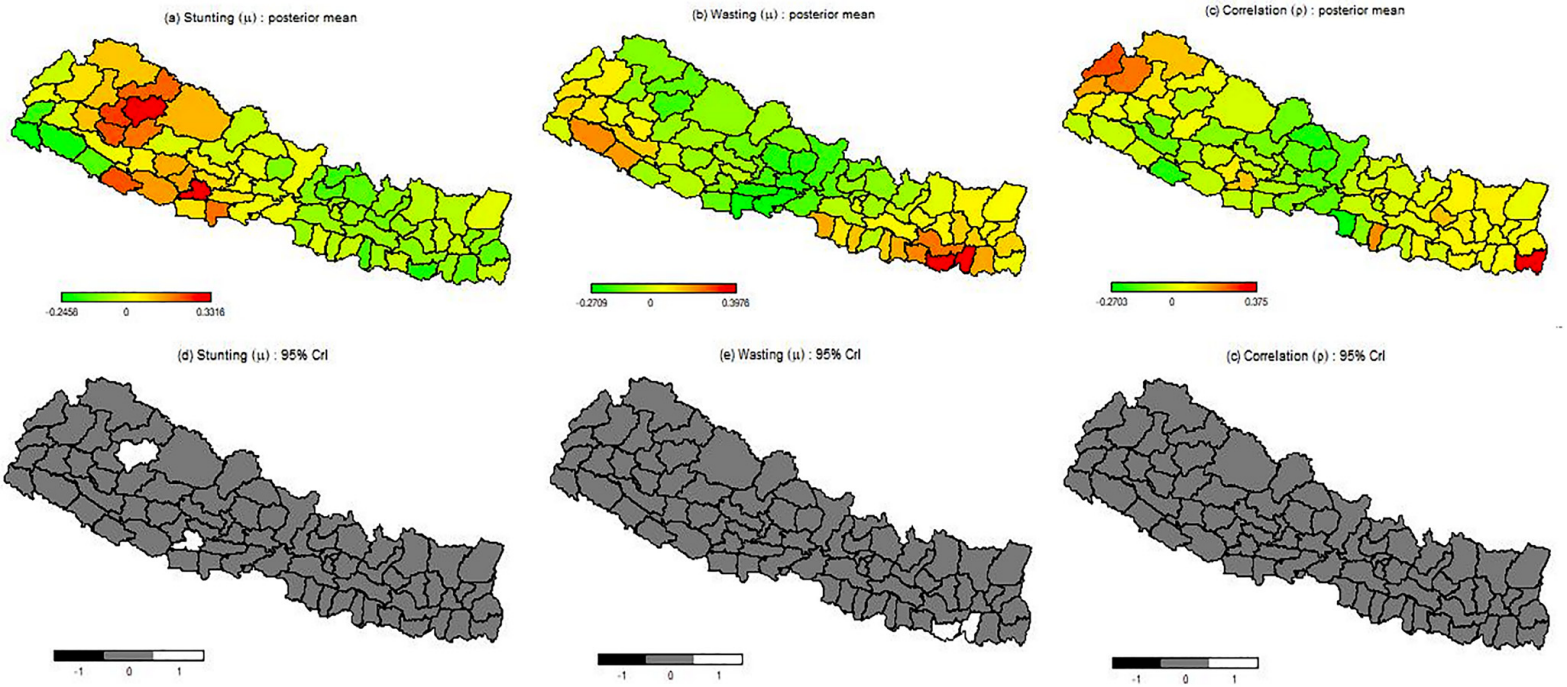
Spatial effects (posterior mean and 95 % CrI) of districts on stunting (mu), wasting (mu) and correlation (rho).

Fig. 2 represents the posterior estimates of district-level spatial effects with top panel maps showing posterior mean and bottom panel indicating the significance of the effects through 95 % credible intervals. In the top panel, yellow to dark red colours on the maps represent positive mean effects, whereas negative effects are shown in yellow to green colours. Grey colour on the bottom panel maps indicates no significance; white colour specifies significant positive mean effects and black points to significant negative mean impacts. The results indicate that children from two districts – one in Karnali province and another in Lumbini province – were significantly more likely to be stunted. In contrast, children from two districts, one in Madhesh and Province 1, were significantly more likely to be wasted. Nevertheless, estimates for spatial effects for correlation were found to be insignificant.

## Discussion

The present study estimates the joint probability of children in Nepal being stunted and wasted, using the distributional bivariate probit model. This is the first work to examine effects of different covariates on stunting and wasting separately and jointly using the correlation of two the outcomes. This approach is assumed to help explore the possible correlation between stunting and wasting and their covariates. This is particularly useful in a resource-scarce country like Nepal where interventions can be prioritised based on the co-existence of the two most common forms of childhood undernutrition. Employing the spatial analysis from the probit model, the study's findings underscore location-specific intervention strategies to address childhood undernutrition. The present study showed that children residing in the districts of Lumbini Province and Karnali Province were more likely to be stunted. Similarly, children from Terai regions in Madhesh Province and Province 1 were more likely to be wasted.

An obvious finding of our study suggests that children from the richest households were less likely to be stunted. Similarly, children from severely food insecure households were more likely to suffer both stunting and wasting. In contrast, children from poorer households were less likely to be stunted and wasted simultaneously, whereas no association was observed for other wealth quintiles. Household wealth and childhood nutritional status are intricately linked. A cohort study involving a pooled analysis of children from four countries reported a significant association between low levels of household wealth and stunting in all four countries. Nonetheless, the association between wasting and household wealth was found to be significant for only one country^(31)^. However, another study from Nepal using a conventional analysis method reported no significant association between wasting and wealth quintile^(32)^. Poorer households are mostly food insecure, such that all household members do not meet the nutritional requirements. Children from food insecure households do not get optimum nutrition, leading to short and long-term nutritional deficiencies^(33)^. Poverty indirectly affects a household's nutritional status. Food insecurity largely affects the health of under-five children in the long-term due to lack of adequate food because the interval between birth to 5 years of age is considered the prime period to attain optimal physical and mental development^(34)^. Nutrition intervention programs should focus on supporting families not able to meet their daily nutritional needs through food subsistence programs. Improving household food insecurity might not be a long-term intervention program, but it can be integrated with other interventions such as improving IYCF practices, safe hygiene and sanitation practices, encouraging families to adopt home-gardening and educating mothers on nutrition-rich foods which have been a successful approach in Nepal^(35,36)^.

Our study reported that a child's low birth weight was associated with children's chronic undernutrition. A child's weight at birth below 2500 g is considered low birth weight and could pose long- and short-term risks to child morbidities and mortality. The World Health Assembly Resolution 2012 endorsed six global nutrition targets for 2025, one of which is a 30 % reduction in low birth weight^(37)^. Reducing low birth weight and preventing the risk of childhood undernutrition requires comprehensive planning, such as improving household food security, educating mothers, enhancing nutrition during pregnancy and care for pregnancy-related conditions, such as improving access to maternal care, pre-eclampsia and social support^(38,39)^. Similarly, our study reported that being a fourth or higher birth order increases the likelihood of stunting in children under the age of five. This is plausible as more children in the household require a higher quantity of diverse food and higher duration of caring time by mothers or caretakers, which are often unmet, especially by poorer and rural Nepalese households. These households are less likely to meet the requirements due to insufficient food production, lower economic status and a longer time spent by mothers and caretakers in household chores and subsistence agricultural work rather than feeding and caring for children.

The findings from our study support that common childhood illnesses, such as fever in the 2 weeks preceding the survey, are associated with stunting in children. Studies have suggested that severe infections during early infancy directly hinder linear growth and cause acute malnutrition^(1,40,41)^. The bidirectional relationship between child nutrition and infection has been widely studied. Frequent infections in children impair their nutritional status, and poor nutrition leads to the risk of disease. Adequate nutrition is required in the body to aid the immune system in fighting against invading organisms^(42)^. Due to this fact, children residing in areas with a high prevalence of common childhood illnesses such as gastrointestinal infections, respiratory illnesses and malaria have low nutritional status.

The linear effect on wasting was significant for currently breastfeeding children in our study. On the contrary, the linear effect of currently breastfeeding on stunting was not significant. Many studies have reported the benefits of breastfeeding, including developing immunity and protection from infection in children and providing optimal nutrition for their overall growth^(43,44)^. However, our findings suggested that wasting is still likely to occur when the children are breastfeeding. Despite the fact that a large majority (89 %) of the children in this study were still breastfed at age two, the literature suggested that the frequency of breastfeeding and quantity of breastmilk consumed, and complementary feeding practices should also be studied when determining the child nutritional status^(19)^.

Childhood undernutrition is highly sensitive to child's age as well as mothers’ age. As suggested by the non-linear effect of child's age on the likelihood of childhood stunting, the likelihood of childhood stunting increases up until 29 months of age and decreases thereafter. The findings also showed that the likelihood of children being wasted decreased sharply from birth to around 30 months of age and increased rapidly thereafter until 59 months. This finding is plausible because 55 % of children in Nepal initiate early breastfeeding and continue to do so through 24 months of age^(45)^. The introduction of semi-solid food is common in Nepal in the fifth or sixth month after childbirth. As the children grow older, their nutritional requirements need to be fulfilled by introducing complementary foods rich in protein for their optimal growth, which might not have been addressed. Similarly, as a part of the National Nutrition Program in Nepal, the Infant and Young Child Feeding (IYCF) program is one of the successful programs in Nepal investing in improving child nutrition of under 2 years of age. But there is a paucity of programs that focus on child nutrition from 24 to 59 months children. Moreover, several socio-demographic factors and cultural practices correlate with child feeding practices among children in Nepal^(46)^. Furthermore, children of an underweight mother were significantly more likely to be stunted or wasted; however, children of overweight mothers had significantly fewer chances of being stunted. Mother's nutritional status is an important determinant of child's nutritional status. Adequate nutrition helps rebuild the body structure of mothers who have recently given birth and aids in the production of milk. The concentration of several micronutrients in breastmilk including Vitamin A, iodine, thiamine and so on depends upon maternal nutritional intake^(47)^. Hence, optimal maternal nutritional status aid children in getting optimal nutrition^(48)^, thereby preventing infections and consequently undernutrition.

The findings of the spatial effects in this study showed a higher likelihood of stunting among children residing in the Lumbini and Karnali provinces. The prevalence of household food insecurity in Karnali and Sudurpaschim provinces was more than 60 %, and food insufficiency for children and lactating mothers poses a greater risk as their nutritional requirement is higher^(19,49)^. Food supply in that region is affected due to hard geographic terrains and lack of road connectivity in most districts. Though there has been some improvement in access to markets and road networks, the food production is not enough to feed people living in the mountain region, where the stunting rate is the highest compared with hills. National policy should prioritise improving household food security in severely food insecure districts to improve nutritional status among children and women. Mothers and children are affected mostly by several nutritional deficiencies. Similarly, the results from the spatial effects suggest that children from Teria districts of Madhesh Province and Province 1 were more likely to be wasted. A study reported that 18⋅9 % children in Eastern Terai region suffered from wasting which is highest compared with other regions of Nepal^(50)^. High rates of wasting in Eastern Terai can be correlated with low energy intake and frequent infections. These findings are consistent with studies reporting similar results from that region^(8,19,32)^. Districts with high rates of stunting and wasting all have poor Human Development Index (HDI) scores and lag considerably behind socio-demographic and health-related indicators^(51)^. This scenario necessitates a critical policy priority as well as region-specific approaches and interventions to lessen the burden of child malnutrition.

The strengths of the present study include the adoption of a method appropriate to represent a joint correlation between acute and chronic malnutrition. This helps determine the factors contributing to the co-existence of both stunting and wasting for designing the region-specific tailored interventions. The findings of the study represent children from across Nepal and can be generalised further as the survey covered all provinces of Nepal. The study also has limitations, such as the inability to measure the role of variables such as dietary intake among under-five children due to the limited number of variables in the main survey itself. Similarly, as the data collected through interviews included events that occurred as long as years ago, the responses might have suffered recall bias. The responses from the interviewees might have suffered social desirability bias as well.

## Implications and further research

The findings of the present study provide several lessons for implementing childhood nutrition programs in Nepal. Firstly, the implementation of childhood nutrition programs should prioritise the geographic regions where the joint burden of acute and chronic malnutrition is high. Secondly, as there is a lack of nutrition programs for children aged 24 months or older, nutrition-sensitive and nutrition-specific programs must be designed and implemented for the age group. A nutrition program alone might not address the burden of overall malnutrition in Nepal, where the socio-demographic determinants, norms and taboos weigh more than improving child nutrition. Hence, childhood nutrition programs and sensitising nutrition enhancement programs using social stakeholders, educating expecting women and recently delivered mothers and organising social regular nutrition-related events would be effective.

## Conclusion

The present study findings highlighted that stunting and wasting among children from poorer households and severely food insecure families is a significant public health problem in Nepal. Our findings underscore a targeted nutritional intervention to deal with undernutrition in the districts of Madhesh, Lumbini and Karnali in Nepal. Implementation of the program should concentrate on improving child undernutrition at the provincial and municipal levels. In addition, further research supported with appropriate statistical modelling are recommended to better understand the burden of childhood undernutrition at provincial and sub-regional levels.
